# Editorial: Functional Annotation of Animal Genomes

**DOI:** 10.3389/fgene.2021.768626

**Published:** 2021-10-15

**Authors:** Amanda Jane Chamberlain, Hans H. Cheng, Elisabetta Giuffra, Christa Kuehn, Christopher K. Tuggle, Huaijun Zhou

**Affiliations:** ^1^ Agriculture Victoria, Agribio, Centre for AgriBiosciences, Bundoora, VIC, Australia; ^2^ Avian Disease and Oncology Laboratory, Washington D.C., MI, United States; ^3^ Paris-Saclay University, INRAE, AgroParisTech, GABI, Jouy-en-Josas, Jouy-en-Josas, France; ^4^ Research Institute for Farm Animal Biology (FBN), Dummerstorf, Germany; ^5^ Iowa State University, Ames, IA, United States; ^6^ University of California Davis, Davis, CA, United States

**Keywords:** genome annotation, farm animals, genomics, sequencing, bioinformatics, resources

All fields of biology have been greatly influenced by the generation of complete and well-annotated genome assemblies. This impact is most apparent with the findings and resulting applications from the Human Genome Project (HGP), which has transformed biomedical science. The original justification for having a genome assembly was to get a complete “parts list” with the primary goal being the identification and location of all genes. However, it soon became readily apparent that genomes were much more than just sequences that code for proteins; protein-coding regions account for ∼1.5% of the human genome and similar results were obtained in analyzing the genomes of domesticated and other farmed animal species. Thus, current efforts have been focused on finding relevant functional elements, such as non-coding elements that regulate when, where, and how much specific genes and/or particular isoforms are expressed.

To address the need for annotation of farm animal genomes, the Functional Annotation of Animal Genomes (FAANG) Consortium was launched in 2015. Like other research consortia, FAANG (www.faang.org) is committed to sharing data rapidly and before publication for the benefit of the whole community (www.faang.org/data-share-principle), with data and metadata (standardized details on samples, laboratory and bioinformatic protocols applied with a comprehensiveness more than current practice) being collected in the FAANG Data Portal (https://data.faang.org/home).

A Research Topic call for papers was made to provide the opportunity to report on the ongoing efforts to annotate farm animal genomes and inform genomic biology. We believed that such a Research Topic would be timely as a historical marker of such efforts, as the pilot FAANG projects were being completed and a number of larger-scale projects are underway in Australia, the United States and Europe. Many groups responded to this call. The Research Topic also offered the opportunity to establish reference-settings for FAANG with respect to methods and protocols. We are pleased that 21 papers, representing eight species as well as two species-agnostic resource reports, are presented in this collection. Below, we summarize reports with complementary themes, focused on one or more of the following topics:

## New Biological and Bioinformatic Resources for the Community

As a community, it is important to have shared resources to minimize duplication of effort, standardize wet-lab protocols, and consistent and readily-available bioinformatic pipelines. Such efforts are a hallmark of the FAANG community from its inception, and several groups reported the completion of different community resources. An European-US effort to describe tissue samples, as well as sample collection protocols and associated metadata across two early FAANG pilot projects, was provided by Tixier-Boichard et al. Several groups reported equine community resources. Donnelley et al. highlighted development of a stallion tissue biobank, a community collaboration to “sponsor” individual tissues to expand epigenetics data for mares was described (Kingsley et al.), and documentation of protocols for measuring chromatin accessible sites using ATAC-seq of equine tissues was contributed (Peng et al.). Two groups outlined FAANG community data resources, including a description of available livestock data and annotation tools at Ensembl (Martin et al.) and an account of the current status and resources available at the FAANG data portal (Harrison et al.).

## Reference Transcriptomes for Cataloging Function and Predicting Regulatory Relationships

Transcriptomic resources are very much akin to the generation of a reference genome assembly, by providing important baseline functional knowledge for highly relevant tissues of each species. Given the continuous improvement in sequencing technologies, it was not surprising that many papers utilized the latest platforms (e.g., long-read sequencing, single cell RNA-seq) to define RNA transcripts and splice variants, as well as chromatin accessibility and epigenetic modifications at RNA-expressing genes. These efforts reflect the breadth of the community in targeting many farmed species spanning fish, birds and mammals for different tissues, developmental stages, and cell types. For example, RNA-seq-based transcriptomes for 10 tissues or isolated cell populations from chickens was summarized by Overbey et al. Iso-seq-based (Chang et al.) and Nanopore-based (Halstead et al.) transcriptomes of a large number of cattle tissues was generated and used to identify full-length transcripts and alternative splicing isoforms, which were often tissue-specifically expressed. Long-read transcriptome technology was also used by Ali et al. on fourteen tissues to improve the annotation of the Rainbow trout genome, as well as identify splice isoforms associated with traits of economic importance in aquaculture. Similar long-read transcriptome analysis of several tissues in Salmon genome annotation was shown to substantially improve the transcript catalog for this species (Ramberg et al.). A new RNA isoform, circular RNA, was cataloged across public and new RNA-seq datasets for three tissues from sheep, cattle, and pig (Robic et al.), and both tissue- and developmental stage-associated differences in abundance of circular RNAs was detected. Further, RNA-seq analysis of eight specific flow-sorted populations of peripheral blood mononuclear cells (PBMC) was compared to single-cell RNA-seq analysis of PBMC by Herrera-Uribe et al. Both datatypes extended annotation for the pig genome, identified co-expressed genes for all major PBMC types in porcine blood, and showed many specific cell types could be matched to human PBMC cell-specific transcriptomes. Finally, co-expression analysis between RNAs and miRNAs across different stages of spermatogenesis was used to predict miRNA regulatory targets in this important process (de Lima et al.).

## Large Scale Functional Annotations: Insights From the Ovine and Caprine FAANG Projects

Chromatin accessibility patterns and epigenomic modifications were reported as outcomes of the ovine FAANG project. The work from Davenport et al. and Massa et al. is setting high standards for analyzing histone modification, transcription factor binding and/or whole genome-wide methylation analyses. The authors demonstrated that the level of activity at the functional genomic elements found correlated with nearby transcriptomic expression. Further exploration of transcription start sites (Salavati et al.) confirmed the spatial association of active genomic elements and initiation of transcription. Furthermore, E and colleagues used whole genome sequencing and Hi-C to provide mechanistic insights as to the biological basis for polled intersex syndrome (PIS) leads to reproductive disorders in goats.

## The Use of Functional Genomic Data to Predict Causal Variants

An ultimate goal of both basic and applied genomics is to connect genotype to phenotype, and multiple groups reported progress in linking genetic variation with the molecular phenotype of RNA expression, which has seen substantial advancement in Genotype-Tissue Expression (GTEx) studies in humans and model species. By analyzing chicken tissues for which both RNA-seq and genomic DNA sequence were available in two populations, Jehl et al. developed thresholds for variant calling and showed the value of existing RNA-seq datasets for reliable SNP detection in allele-specific expression (ASE) and future GTEx studies. In a second report from this group and again investigating chicken RNA-seq data, Degalez et al. reported on the value of haplotype-aware variant annotation and the interest to consider multi-nucleotide variants in the coding regions. Prowse-Wilkins et al. produced and integrated histone modification and CTCF data across six tissues from lactating dairy cows to identify partitions of the genome predicted to comprise functional regions in these tissues. Importantly, they then showed the level of activity of these functional regions were correlated with nearby gene expression and such regions were enriched for putative causal variants. Interestingly, the level of enrichment improved where regions were correlated with the level of expression and was greatest for QTL for milk production traits. This work provided strong evidence for the core hypothesis of the FAANG project; that form follows function and cataloging genome functional elements can be used to find important (e.g., predictive) variation likely causing phenotypic differences.

As exemplified by this collection, the efforts produced by groups throughout the world indicate the future of FAANG is very bright. Having said this, the value of the insights provided by the currently more comprehensive efforts in human and biomedical models is clear, and significantly more progress will be needed to fully exploit the public investment in animal agricultural genomics. Especially challenging will be the validation of predicted functional elements and the verification of casual variants associated with complex traits, as each polymorphism may have only a small effect. However, the next major advancements in translation of farm animal genome functional variation into prediction of biological phenotype will come from such precise knowledge of individual genomes.

We close by congratulating each of the contributing authors for their outstanding work, and extend our appreciation to all of the reviewers for their time and effort to improve each submission.

